# Pretreatment Inflammatory Indices as Predictors of Survival in Endometrial Cancer: A Retrospective Cohort Study From Mexico

**DOI:** 10.7759/cureus.89855

**Published:** 2025-08-12

**Authors:** Cintia M Sepúlveda-Rivera, Pamela L Rico-Mejía, Maria D Pérez-Montiel, David Cantú-de León, Rosa A Salcedo-Hernández, Pamela Martínez-Alpizar, Sebastian Duno-Caldera, Salim A Barquet-Muñoz

**Affiliations:** 1 Gynecologic Oncology, Instituto Nacional de Perinatología, Mexico City, MEX; 2 Gynecologic Oncology, Instituto Nacional de Cancerología, Mexico City, MEX; 3 Pathology, Instituto Nacional de Cancerología, Mexico City, MEX; 4 Surgical Oncology, Instituto Nacional de Cancerología, Mexico City, MEX; 5 Gynecology, Instituto Nacional de Perinatología, Mexico City, MEX

**Keywords:** endometrial cancer (ec), inflammatory indices, lymphocyte-to-monocyte ratio, overall survival (os), prognostic nutritional index

## Abstract

Introduction: Nutritional and inflammatory prognostic indices derived from biochemical and hematological parameters are emerging as potential prognostic biomarkers in various malignancies, including endometrial cancer.

Objective: The objective of this study is to evaluate whether pre-treatment biomarkers of systemic inflammation are associated with survival outcomes in patients with endometrial cancer.

Methods: This retrospective cohort study included women treated for endometrial cancer at a single institution. Pre-treatment Onodera’s Prognostic Nutritional Index (PNI), Neutrophil-to-Lymphocyte Ratio (NLR), Platelet-to-Lymphocyte Ratio (PLR), Lymphocyte-to-Monocyte Ratio (LMR), and Systemic Immune-Inflammation Index (SIII) were calculated, with cutoff values determined using receiver operating characteristic (ROC) curves and quartiles. Associations with overall survival (OS) were analyzed using Kaplan-Meier estimation and multivariable Cox proportional regression, adjusting for the International Federation of Gynecology and Obstetrics (FIGO) stage, histology, grade, and adjuvant therapy. Odds ratios (ORs) were used due to the retrospective design.

Results: We included 887 patients who underwent initial staging surgery between 1997 and 2019. The median age was 55.27 years, with a median follow-up of 59.63 months. High NLR (>2.8, OR 1.90, 95% confidence interval (CI): 1.26-2.87, p=0.002) and PLR (>103.2) and low LMR (<4.8, OR 2.09, 95% CI 1.39-3.15, p<0.001, area under the curve (AUC) 0.589, 95% CI 0.54-0.64) and PNI (<40.0) were associated with reduced five-year OS. The LMR demonstrated the highest, though modest, precision in AUC analysis and was significantly associated with OS and mortality risk. These indices may guide adjuvant therapy decisions in resource-limited settings.

Conclusion: Pre-treatment inflammatory indices, particularly LMR, are promising prognostic biomarkers for OS in endometrial cancer, offering accessible tools for risk stratification in low-middle-income settings. These indices should not replace established clinical prognosticators but may provide additive value. Prospective, multicenter studies are needed to confirm these findings and integrate them into clinical practice.

## Introduction

Endometrial cancer is the sixth most common cancer globally, with an estimated 420,000 new cases in 2024 [1]. Its incidence in Mexico has risen over the past decade [2]. Approximately 75% of cases are diagnosed at early stages [3], with surgery as the primary treatment [4]. Early intervention often leads to prolonged survival, making treatment-related morbidity a critical consideration.

Clinicopathological factors, such as age, histological subtype, stage, grade, and the presence of cervical stromal, lymphovascular, or myometrial invasion, as well as lymph node involvement, are commonly used for risk stratification; however, most of these variables are typically assessed postoperatively [4]. Thus, identifying pretreatment biomarkers that can accurately predict prognosis is essential.

Emerging evidence suggests that systemic inflammation influences endometrial cancer prognosis. Chronic inflammation, driven by adipose tissue expansion and hypoxia, fosters a proinflammatory environment with elevated cytokines, interferons, interleukins, and C-reactive protein [5]. This promotes cell proliferation and inhibits apoptosis, contributing to tumor growth [6]. Pretreatment inflammatory indices, calculated from routine blood tests, reflect immune surveillance (e.g., lymphocyte-mediated antitumor responses) versus tumor-promoting myeloid responses (e.g., monocytes differentiating into tumor-associated macrophages) [7].

We evaluated five indices: Neutrophil-to-Lymphocyte Ratio (NLR, neutrophils ÷ lymphocytes), Platelet-to-Lymphocyte Ratio (PLR, platelets ÷ lymphocytes), Lymphocyte-to-Monocyte Ratio (LMR, lymphocytes ÷ monocytes), Onodera’s Prognostic Nutritional Index (PNI, 10 × albumin (g/L) + 0.005 × lymphocyte count), and Systemic Immune-Inflammation Index (SIII, (platelets × neutrophils) ÷ lymphocytes). These were selected based on prior evidence in endometrial and gynecologic cancers, demonstrating associations with survival and disease progression [8-14]. For example, PNI has been validated as a prognostic factor in gynecologic cancers [8], while NLR and PLR predict survival in endometrial cancer [9,10]. LMR and SIII have shown promise in endometrial and other malignancies [11,12]. However, their prognostic utility in endometrial cancer remains underexplored, particularly in low-middle-income settings [13].

## Materials and methods

This retrospective cohort study included all women with histopathologically confirmed endometrial carcinoma treated at the Instituto Nacional de Cancerología (INCan), Mexico City, from 1997 to 2019 who met the inclusion criteria. The study included 887 patients, sufficient to detect significant associations based on prior studies [14,15]. Women aged ≥18 years with endometrial carcinoma who underwent primary surgical treatment (hysterectomy with or without lymphadenectomy) at INCan and had complete pretreatment laboratory data (within 30 days of surgery) were included. Patients were excluded if they had incomplete laboratory data, concurrent or prior second primary malignancy, hematological disorders, surgery outside INCan, or neoadjuvant chemotherapy.

Clinicopathological data and pretreatment biochemical/hematological parameters were retrieved from electronic medical records. Parameters included leukocytes, hemoglobin, platelets, neutrophils, lymphocytes, monocytes, albumin, and CA-125. Inflammatory indices were calculated as described above. Cutoff points were determined using quartiles for NLR (2.8) and PLR (103.2) to align with prior literature [14,15] and receiver operating characteristic (ROC) curves with Youden’s index for LMR (4.8), PNI (40.0), and SIII (895.0) to optimize sensitivity and specificity. Missing data led to the exclusion of 172 patients.

The normality of continuous variables was assessed using the Shapiro-Wilk test. Continuous variables were reported as medians with interquartile ranges (IQR) for non-normal distributions or means with standard deviations for normal distributions. Categorical variables were expressed as frequencies and percentages. Comparisons used students' t-test or the Wilcoxon rank-sum test for continuous variables and the chi-square or Fisher’s exact test for categorical variables. Statistical significance was set at p<0.05. Overall survival was analyzed using Kaplan-Meier estimation and multivariable Cox proportional regression, adjusting for the International Federation of Gynecology and Obstetrics (FIGO) stage, histology, grade, and adjuvant therapy. Odds ratios were used instead of hazard ratios due to the retrospective design, which limits time-to-event analysis. Subgroup analyses by FIGO stage (I-II vs. III-IV) and histology (endometrioid vs. high-risk) were performed using Cox regression, adjusting for covariates, to assess differential prognostic effects. All analyses adhered to the 1964 Helsinki Declaration, and the study was approved by the INCan ethics committee (registration number Ref/INCAN/C1/0405/2022).

## Results

Of 1654 patients screened, 767 (46.4%) were excluded (399 (24.1%) underwent surgery outside INCan, 99 (6.0%) did not undergo surgery as initial treatment, 97 (5.9%) had a second malignancy, 172 (10.4%) lacked complete clinicopathological data). The final analysis included 887 patients.

The median age was 55.27 years (IQR 46-63.31). According to the 2009 FIGO staging system, 595 (67.08%) had stage I, 93 (10.48%) stage II, 163 (18.38%) stage III, and 36 (4.06%) stage IV disease. Endometrioid histology predominated (725 (81.74%)). Median inflammatory indices were NLR, 2.0 (IQR 1.55-2.81); PLR, 135 (IQR 103.21-180); LMR, 4.75 (IQR 3.67-6.0); PNI, 40.01 (IQR 38.01-42.01); SIII, 594.75 (IQR 403.58-894.65) (Table 1).

**Table 1:** General characteristics of the patients (n=887). Data are presented as median (interquartile range, IQR) for continuous variables (e.g., age, BMI, inflammatory indices) and n (%) for categorical variables (e.g., menopause, histology, FIGO stage). Statistical significance was set at p<0.05. FIGO: International Federation of Gynecology and Obstetrics (2009 staging); BMI: body mass index; IQR: interquartile range

Characteristic	Value
Age, years, median (IQR)	55.27 (46.0-63.3)
Menopause, n (%)	648 (73.1)
BMI, kg/m^2^, median (IQR)	30.47 (26.3-35.1)
Histology, n (%)	
Endometrioid	725 (81.7)
Papillary serous	41 (4.6)
Clear cells	15 (1.7)
Mixed	73 (8.2)
Carcinosarcoma	27 (3.0)
Others	6 (0.7)
Grade, n (%)	
Grade 1	147 (16.6)
Grade 2	459 (51.7)
Grade 3	119 (13.4)
High-risk histology	162 (18.3)
Myometrial invasion, n (%)	
<50%	583 (65.7)
≥50%	304 (34.3)
Cervical stromal invasion, n (%)	202 (22.8)
Serous involvement, n (%)	35 (3.9)
Adnexal involvement, n (%)	63 (7.1)
Pelvic metastasis, n (%)	
Negative	346 (39.0)
Positive	126 (14.2)
Lymphadenectomy not performed	415 (46.8)
Para-aortic metastasis, n (%)	
Negative	311 (35.1)
Positive	75 (8.5)
Lymphadenectomy not performed	501 (56.5)
Positive lymph nodes	141 (15.9)
Parametrial involvement	23 (2.6)
Lymphovascular space invasion	289 (32.6)
FIGO Stage (2009), n (%)	
I	595 (67.1)
II	93 (10.5)
III	163 (18.4)
IV	36 (4.1)
Adjuvant therapy, n (%)	462 (52.1)
Radiotherapy	443 (95.9)
Chemotherapy	236 (51.1)
Inflammatory indices, median (IQR)	
Neutrophil-to-Lymphocyte Ratio (NLR)	2.0 (1.6-2.8)
Platelet-to-Lymphocyte Ratio (PLR)	135 (103.2-180.0)
Lymphocyte-to-Monocyte Ratio (LMR)	4.8 (3.7-6.0)
Onodera’s Prognostic Nutritional Index (PNI)	40.0 (38.0-42.0)
Systemic Immune-Inflammation Index (SIII)	594.8 (403.6-894.7)
Recurrence, n (%)	127 (14.3)
Mortality, n (%)	118 (13.3)

**Figure 1:**
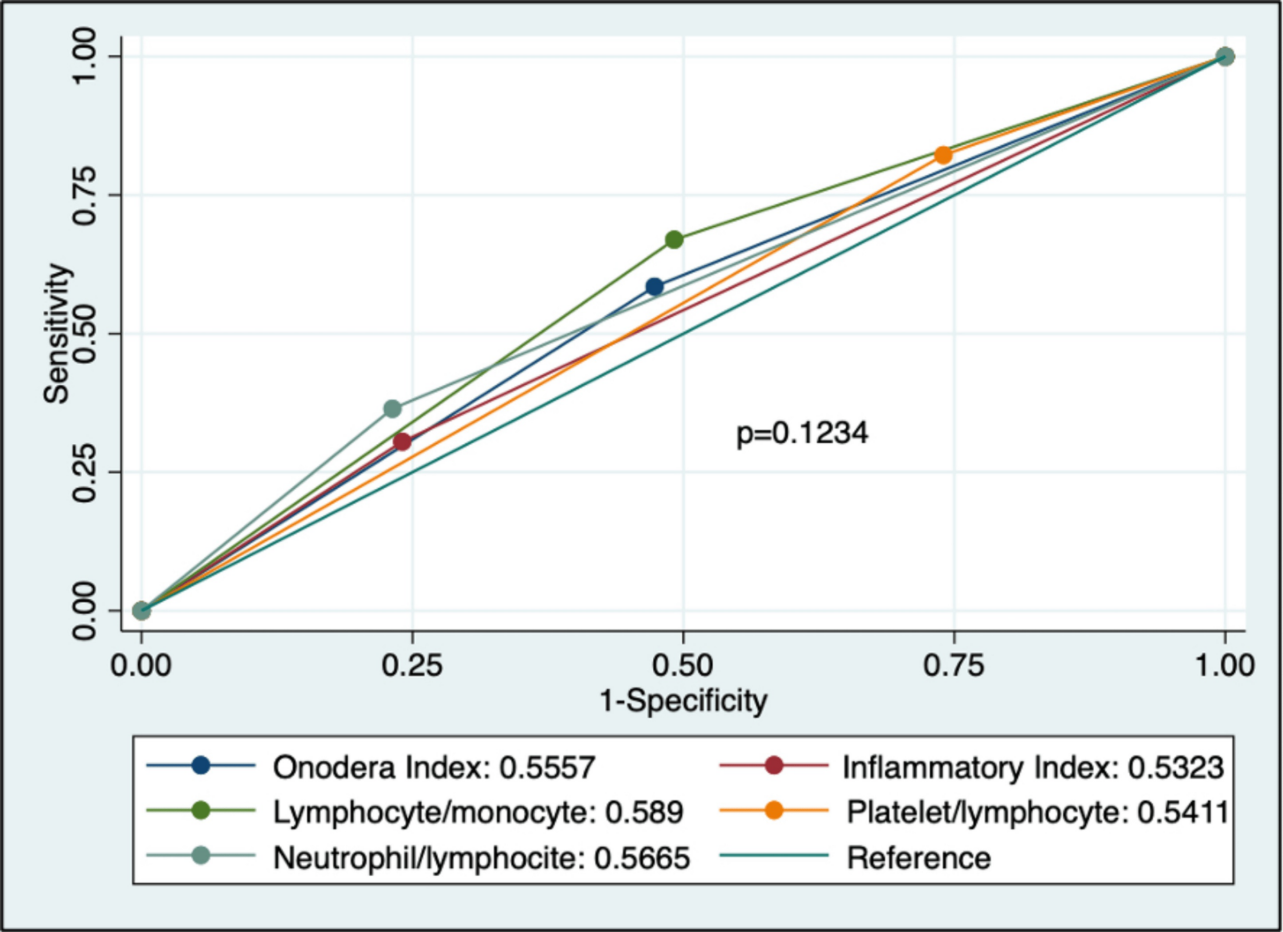
Area under the curve (AUC) for each inflammatory index, presented as numerical values with 95% confidence intervals. Statistical significance was set at p<0.05.

The cut-off points were as follows: NLR 2.8, PLR 103.2, LMR 4.8, PNI 40.0, and SIII 895.0, determined using ROC curves for LMR, PNI, and SIII, and quartiles for NLR and PLR. The diagnostic accuracies of these indices are shown in Table 2. LMR had the highest area under the curve (AUC) (0.589, 95% CI 0.54-0.64), with a sensitivity of 66.95% and specificity of 50.85%, but no significant differences were found between the indices as shown in Figure 1.

**Table 2:** Diagnostic precision of the evaluated indices. Data are presented as percentages for sensitivity and specificity, and numerical values for area under the curve (AUC) with 95% confidence intervals (CI). P-values for AUC comparison were calculated using DeLong’s test. Statistical significance was set at p<0.05.

Inflammatory Index	Sensitivity (%)	Specificity (%)	AUC (95% CI)	Test Statistic (z)	p
Neutrophil-to-Lymphocyte Ratio (NLR)	36.44	76.85	0.567 (0.52-0.61)	z = 1.15	0.123
Platelet-to-Lymphocyte Ratio (PLR)	82.20	26.01	0.540 (0.50-0.58)	z = 0.75	0.453
Lymphocyte-to-Monocyte Ratio (LMR)	66.95	50.85	0.589 (0.54-0.64)	z = 1.89	0.059
Onodera’s Prognostic Nutritional Index (PNI)	58.47	52.67	0.556 (0.51-0.60)	z = 1.26	0.208
Systemic Immune-Inflammation Index (SIII)	30.51	75.94	0.532 (0.49-0.58)	z = 0.64	0.523

**Figure 2:**
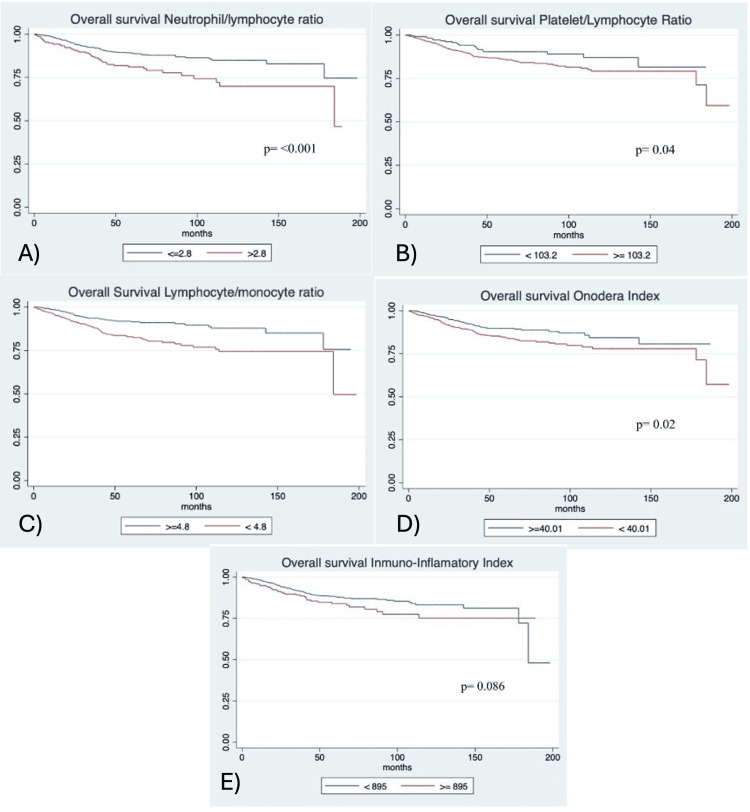
Kaplan-Meier survival curves for each inflammatory index, showing five-year overall survival probabilities as percentages with interquartile ranges (IQR). (A) Neutrophil-to-Lymphocyte Ratio (NLR), (B) Platelet-to-Lymphocyte Ratio (PLR), (C) Lymphocyte-to-Monocyte Ratio (LMR), (D) Onodera’s Prognostic Nutritional Index (PNI), and (E) Systemic Immune-Inflammation Index (SIII). Statistical significance was set at p<0.05.

The median follow-up was 59.63 months, with a five-year OS rate of 89.01% (IQR 86.15-91.32, p<0.001). Survival outcomes by index cutoff points, as shown in Figure 2, were NLR: 91.49% (IQR 88.14-93.92, n=650, p<0.001) for ≤2.8 vs. 81.12% (IQR 74.57-86.13, n=237) for >2.8; PLR: 90.38% (IQR 85.15-93.85, n=222, p=0.046) for <103.2 vs. 86.02% (IQR 82.88-88.63, n=665) for >103.2; LMR: 82.86% (IQR 78.67-86.31, n=444, p<0.001) for <4.8 vs. 91.49% (IQR 88.14-93.92, n=443) for ≥4.8; PNI: 84.27% (IQR 80.02-87.68, n=444, p=0.022) for <40.01 vs. 89.77% (IQR 86.39-92.35, n=443) for ≥40.01; SIII: 88.12% (IQR 85.16-90.52, n=665, p=0.086) for <895 vs. 84.03% (IQR 77.85-88.62, n=222) for ≥895.

Subgroup analyses by FIGO stage revealed stronger LMR associations in stages III-IV (OR 2.45, 95% CI 1.50-4.00, p<0.001) versus stages I-II (OR 1.80, 95% CI 1.10-2.95, p=0.019). High-risk histologies (e.g., serous) showed a stronger LMR effect (OR 2.60, 95% CI 1.45-4.65, p=0.001).

Associations with mortality were as shown in Table 3: NLR: OR 1.90 (95% CI 1.26-2.87, p=0.002); PLR: OR 1.62 (95% CI 0.99-2.67, p=0.057); LMR: OR 2.09 (95% CI 1.39-3.15, p<0.001); PNI: OR 1.57 (95% CI 1.06-2.32, p=0.025); SIII: OR 1.39 (95% CI 0.91-2.12, p=0.133).

**Table 3:** Association analysis of the evaluated indices. Data are presented as odds ratios (OR) with 95% confidence interval (CI), adjusted for FIGO stage, histology, grade, and adjuvant therapy. P-values were calculated using the Wald test. Statistical significance was set at p<0.05. FIGO: International Federation of Gynecology and Obstetrics (2009 staging)

Inflammatory Index	OR	95% CI	Test Statistic (z)	p
Neutrophil-to-Lymphocyte Ratio (NLR)	1.90	1.26-2.87	z = 3.04	0.002
Platelet-to-Lymphocyte Ratio (PLR)	1.62	0.99-2.67	z = 1.90	0.057
Lymphocyte-to-Monocyte Ratio (LMR)	2.09	1.39-3.15	z = 3.55	<0.001
Onodera’s Prognostic Nutritional Index (PNI)	1.57	1.06-2.32	z = 2.24	0.025
Systemic Immune-Inflammation Index (SIII)	1.39	0.91-2.12	z = 1.49	0.133

## Discussion

This study found that high NLR and PLR and low LMR and PNI were associated with reduced five-year OS in endometrial cancer, consistent with prior studies [15,16]. The LMR exhibited the highest, though modest, AUC (0.589, 95% CI 0.54-0.64) and a significant association with OS (OR 2.09, 95% CI 1.39-3.15, p<0.001), suggesting its potential as a prognostic marker. The modest AUCs indicate limited individual predictive power, suggesting these indices should supplement, not replace, clinical staging [4].

The LMR’s prognostic strength may reflect the balance between lymphocytes (antitumor) and monocytes (tumor-promoting) [17]. Eo WK et al. reported that a low LMR (cutoff 3.28) was associated with worse OS (76.0% vs. 62.5%, p=0.001) [18], while Dong et al. found that NLR >2.47 predicted advanced stage and OS [19]. Our findings align, using population-specific cutoffs. Similarly, Muangto et al. demonstrated that a PLR ≥134.95 predicted >50% myometrial invasion with 75.0% sensitivity and 55.6% specificity [20], which is consistent with our results.

The PNI’s association with reduced OS (OR 1.57, p=0.025) supports Njoku et al., who reported a 45% reduction in OS for PNI ≥45 [7]. Our lower cutoff (40.0) may reflect nutritional differences in a low-middle-income population. The SIII’s weaker performance (OR 1.39, p=0.133) contrasts with Huang et al. [21], possibly due to preoperative versus postoperative measurements and variability in inflammatory responses. Subgroup analyses revealed stronger LMR associations in advanced stages (III-IV) and high-risk histologies, suggesting tailored cutoffs may enhance utility.

In resource-limited settings, LMR’s accessibility from routine blood tests is a key advantage. For example, patients with low LMR and stage III-IV disease could be prioritized for aggressive adjuvant therapy, potentially improving outcomes. The 22-year accrual period is appropriate for endometrial cancer due to its often prolonged survival, though advancements in surgical and adjuvant management may introduce heterogeneity. We did not stratify by treatment epoch to maintain focus on the prognostic indices, but this is noted as a limitation.

Strengths include the large sample size, long follow-up, and comprehensive comparison of five indices. Limitations include the retrospective design, single-center data, lack of internal/external validation (e.g., bootstrap resampling), and modest AUCs. The hybrid cutoff approach (quartiles for NLR, ROC for others) may introduce methodological heterogeneity, though justified by literature alignment and optimization.

## Conclusions

Pretreatment inflammatory indices, particularly LMR, are promising prognostic biomarkers for endometrial cancer survival, offering cost-effective risk stratification in low-middle-income settings. These indices should not replace established clinical prognosticators but may provide additive value, such as guiding adjuvant therapy decisions for high-risk patients. The retrospective, single-center design and modest AUCs limit generalizability, necessitating prospective, multicenter validation with standardized cutoffs and validation methods like bootstrap resampling.

## References

[1] Ferlay J, Ervik M, Lam F (2024). Global Cancer Observatory: Cancer Today. https://gco.iarc.who.int/today.

[2] Gallardo-Rincón D, Toledo-Leyva A, Bahena-González A (2021). Validation of the QLQ-EN24 instrument for the assessment of health-related quality of life for women with endometrial cancer in México. Arch Gynecol Obstet.

[3] Siegel RL, Miller KD, Jemal A (2020). Cancer statistics, 2020. CA Cancer J Clin.

[4] Concin N, Matias-Guiu X, Vergote I (2021). ESGO/ESTRO/ESP guidelines for the management of patients with endometrial carcinoma. Int J Gynecol Cancer.

[5] Waldman AD, Fritz JM, Lenardo MJ (2020). A guide to cancer immunotherapy: from T cell basic science to clinical practice. Nat Rev Immunol.

[6] Yang R, Chang Q, Meng X, Gao N, Wang W (2018). Prognostic value of systemic immune-inflammation index in cancer: a meta-analysis. J Cancer.

[7] Njoku K, Barr CE, Ramchander NC, Crosbie EJ (2022). Impact of pre-treatment prognostic nutritional index and the haemoglobin, albumin, lymphocyte and platelet (HALP) score on endometrial cancer survival: a prospective database analysis. PLoS One.

[8] Peng J, Zhang R, Zhao Y (2017). Prognostic value of preoperative prognostic nutritional index and its associations with systemic inflammatory response markers in patients with stage III colon cancer. Chin J Cancer.

[9] Li S, Tian G, Chen Z, Zhuang Y, Li G (2019). Prognostic role of the prognostic nutritional index in pancreatic cancer: a meta-analysis. Nutr Cancer.

[10] Wang X, Wang Y (2019). The prognostic nutritional index is prognostic factor of gynecological cancer: a systematic review and meta-analysis. Int J Surg.

[11] Guo Y, Shi D, Zhang J (2019). The hemoglobin, albumin, lymphocyte, and platelet (HALP) score is a novel significant prognostic factor for patients with metastatic prostate cancer undergoing cytoreductive radical prostatectomy. J Cancer.

[12] Peng D, Zhang CJ, Gong YQ, Hao H, Guan B, Li XS, Zhou LQ (2018). Prognostic significance of HALP (hemoglobin, albumin, lymphocyte and platelet) in patients with bladder cancer after radical cystectomy. Sci Rep.

[13] Lou C, Jin F, Zhao Q, Qi H (2022). Correlation of serum NLR, PLR and HALP with efficacy of neoadjuvant chemotherapy and prognosis of triple-negative breast cancer. Am J Transl Res.

[14] Hu B, Yang XR, Xu Y (2014). Systemic immune-inflammation index predicts prognosis of patients after curative resection for hepatocellular carcinoma. Clin Cancer Res.

[15] Cong R, Kong F, Ma J, Li Q, Wu Q, Ma X (2020). Combination of preoperative neutrophil-lymphocyte ratio, platelet-lymphocyte ratio and monocyte-lymphocyte ratio: a superior prognostic factor of endometrial cancer. BMC Cancer.

[16] Cummings M, Merone L, Keeble C (2015). Preoperative neutrophil:lymphocyte and platelet:lymphocyte ratios predict endometrial cancer survival. Br J Cancer.

[17] Li J, Jiang R, Liu WS (2013). A large cohort study reveals the association of elevated peripheral blood lymphocyte-to-monocyte ratio with favorable prognosis in nasopharyngeal carcinoma. PLoS One.

[18] Eo WK, Kwon S, Koh SB (2016). The lymphocyte-monocyte ratio predicts patient survival and aggressiveness of endometrial cancer. J Cancer.

[19] Dong Y, Cheng Y, Wang J (2019). The ratio of neutrophil to lymphocyte is a predictor in endometrial cancer. Open Life Sci.

[20] Muangto T, Maireang K, Poomtavorn Y (2022). Study on preoperative neutrophil/lymphocyte (NLR) and platelet/lymphocyte ratio (PLR) as a predictive factor in endometrial cancer. Asian Pac J Cancer Prev.

[21] Huang Y, Chen Y, Zhu Y, Wu Q, Yao C, Xia H, Li C (2021). Postoperative systemic immune-inflammation index (SII): a superior prognostic factor of endometrial cancer. Front Surg.

